# COVID-19 pandemic and social distancing: economic, psychological, family, and technological effects

**DOI:** 10.47626/2237-6089-2020-0085

**Published:** 2021-05-08

**Authors:** Luciane Maria Both, Gustavo Zoratto, Vitor Crestani Calegaro, Luis Francisco Ramos-Lima, Bianca Lorenzi Negretto, Simone Hauck, Lucia Helena Machado Freitas

**Affiliations:** 1 Programa de Pós-Graduação em Psiquiatria e Ciências do ComportamentoUniversidade Federal do Rio Grande do SulPorto AlegreRSBrazil Programa de Pós-Graduação em Psiquiatria e Ciências do Comportamento , Universidade Federal do Rio Grande do Sul (UFRGS), Porto Alegre , RS . Brazil .; 2 Faculdade de MedicinaUniversidade Federal de Santa MariaSanta MariaRSBrazil Faculdade de Medicina , Universidade Federal de Santa Maria (UFSM), Santa Maria , RS , Brazil .; 3 Departamento de NeuropsiquiatriaUFSMSanta MariaRSBrazil Departamento de Neuropsiquiatria , UFSM , Santa Maria , RS , Brazil .; 4 Programa de Pesquisa e Tratamento de Trauma PsicológicoHospital de Clínicas de Porto AlegrePorto AlegreRSBrazil Programa de Pesquisa e Tratamento de Trauma Psicológico (NET-Trauma), Hospital de Clínicas de Porto Alegre (HCPA), Porto Alegre , RS , Brazil .

**Keywords:** Coronavirus, COVID-19, pandemic, social distance

## Abstract

**Introduction:**

The concept of social isolation is currently understood as a measure of epidemiological containment that aims to reduce the speed of spread of the disease, enabling health services to prepare their resources to cope with the likely increase in demand, while also seeking to provide additional protection to groups considered to be at higher risk.

**Objective:**

The present narrative review aims to compile and synthesize the literature related to social isolation produced during the COVID-19 pandemic in 2020.

**Method:**

This study is a narrative review of the literature on social isolation in the context of the COVID-19 pandemic.

**Results:**

73 publications were included for full-text reading and were classified into the following categories: levels of social isolation, economic effects, family relationships, health system, mental health of the population, and use of technology.

**Conclusions:**

It is necessary to plan an escalation of responses to the consequences of the pandemic, especially in view of the increased demand on the health sector and social services. The negative effects of social isolation can be prevented by public policies that offer a response to the economic recession, maintenance of social work, encouragement of quality care in mental health services, and community support for vulnerable families.

## Introduction

The COVID-19 pandemic started in December 2019 in Wuhan (China), with the emergence of a specific coronavirus, known as SARS-CoV-2. Worldwide spread of the virus reached pandemic severity in March 2020, as declared by the World Health Organization. ^1^ The first confirmed case in Brazil was registered on February 26, 2020, in the state of São Paulo ^2^ and, since then, the number of cases and deaths due to COVID-19 has been increasing daily. Thus, in order to contain the overwhelming dissemination, certain technical safety precautions have been adopted to take a more stringent approach to public health protection. Such containment measures have been adopted in all countries involved ^3 , 4^ and include mandatory use of face masks, implementation of social distancing, cancellation of events (conferences, sports competitions), strict travel restrictions, closure schools/universities and most of the workplaces (except essential health services, press, food and primary assets). On the other hand, some countries were slow to carry out epidemic control measures, suffering the consequences of vacillation by central authorities. ^5^

Social isolation includes measures such as avoiding contact with family and friends, preference for home delivery of essential items, and reduction of social coexistence, which when it does take place, should observe a minimum distance of two meters between people. ^6^ Although social isolation is effective as a public health measure to contain viral spread, in psychological terms it can arouse fears, uncertainties, and despair. ^7 , 8^ Additionally, the impact on the economy and health systems, changes to family relationships, and migration to unprecedented levels of technology use are all happening simultaneously. The pandemic constitutes an urgent situation and demands a range of quick and effective responses from people. Therefore, rapid and systematic efforts are being made in research and clinical settings, mainly in the academic environment and on the so-called “front line”, as demonstrated by the increasing numbers of publications on the subject over recent months. One of the goals of these efforts is to employ different methods to understand the effects of this new external situation on the internal situation, while other targets of investigation include the emergence or worsening of psychiatric symptoms. Prompt dissemination of the findings should contribute knowledge on how to better care for people in general. ^9^

Against this background, this study aims to review the literature related to social isolation produced during the COVID-19 pandemic in 2020. It is expected that analysis of this scientific production and its synthesis in this article will provide a concise and well-founded source to be read by professionals involved in the care of people in isolation.

## Method

This study is a narrative review of the literature on social isolation in the context of the COVID-19 pandemic. The descriptors “pandemic”, “COVID-19” and “social isolation” were used to search the CAPES Periódicos (a Brazilian database), PubMed, SciELO, and Google Scholar databases. All studies published during 2020 in Portuguese or English up to May 31, 2020 were included in the analysis, in addition to brief communications and theoretical reflections. After reading the search results, three publications that did not mention social isolation during the COVID-19 pandemic were excluded. Data were compiled into analytical categories defined by the authors a posteriori, to better understand aspects related to isolation in the context of the pandemic, covering them in depth.

## Results

After a careful perusal of the abstracts identified, 73 publications were included for full-text reading and were classified into the following categories: levels of social isolation, economic effects, family relationships, health system, mental health of the population, and use of technology.

### Socioeconomic aspects

In this category we integrate issues related to levels of social isolation, economic effects, and health systems.

#### Levels of social isolation

Non-pharmaceutical isolation policies, such as social distancing, have been proven to be the only possible response to stop the spread of the virus when it comes to a pandemic. ^10 , 11^ However, different strategies for centralized and decentralized social distancing exist. Centralized strategies refer to governments granting tax relief and tax holidays; whereas a decentralized strategy is restricted to conscious isolation (neighbors of affected children isolate themselves from those infected) and self-isolation (individuals aware of their illness sever social ties in their ego network). ^12^ These different degrees of social distance are crucial to reducing the peak rate of contamination. Data show that moderate social distance (cutting 50% of social ties) can reduce the impact of the outbreak by 47% for centralized isolation and by 31% for decentralized isolation. Countries that decided to implement a reactive response to the pandemic with immediate adoption of isolation measures showed less damage from infection. ^12^

There seems to be a need for distinct measures aimed at high-risk groups to decrease mortality in this population; such measures require mobility restrictions (provision of food, medicines, and other essential items at home) and increased support to meet communication and medical care needs. ^10^ The laxity of social isolation strategies needs to take into account that, although some people remain voluntarily or habitually at a social distance, others seek high levels of social reengagement as soon as possible. ^13^

Different countries follow different combinations of social distance. For example, in Sweden, only moderate social distancing has been implemented. This strategy targets the possibility of developing the population’s immunity, but it can generate high mortality and saturation of the health system, since there are no specific measures for the vulnerable population. Italy and Spain implemented uniform strict social distancing, but even so, this strategy was unable to stop the pandemic from advancing, perhaps because of the physical proximity between generations of elderly and young people or because it was executed late. In Germany, where different isolation measures were adopted for vulnerable people, the mortality rate was lower. ^10^

Social isolation was not seen as a positive measure in some countries. For example, in Hubei, China, the population revolted against the isolation imposed by the government ^14^ ; while in Jordan it caused chaos in the population. ^15^

Data obtained in a Chinese province with 1925 participants showed that the measures of social isolation implemented by local governments must take into account the theory of planned behavior, which identifies three factors related to the desire to isolate oneself in the face of a pandemic emergency: attitudes (degree to which a person has an unfavorable assessment of the behavior in question), subjective norms (perceived social pressure to perform a behavior or not), and perceived control (perceived ease or difficulty in executing the behavior and is assumed to reflect past experience, as well as anticipated obstacles). The attitude factor was the most relevant, referring to the degree to which the person has a favorable or unfavorable assessment of social isolation. ^16^

#### Economic effects

One relevant issue of the pandemic is the great economic impact on families’ incomes, because of restrictions on establishments’ operations. Other social vulnerabilities are unleashed as a consequence. ^17^ The economic effects of the pandemic are evident: closing workplaces (whether for the employee or the owner of the establishment), closing schools (which may require comprehensive childcare on the part of families or hiring someone extra to assist in this role), and the threat to housing security with non-payment of rent or real estate loans, ^3^ increasing the risk of homelessness. ^11^ A survey of 27 participants in the UK found that social isolation caused a fall in pay and even loss of income. ^13^ Nevertheless, in spite of the predominantly negative scenario, the long stay at home can reconfigure our consumption habits, making people more sustainable, frugal, and responsible in terms of use of resources and of finding ways of reusing them creatively. ^5^

The pandemic always affects the most vulnerable people the most violently: street people, slum dwellers, and others. These individuals are isolated in extremely unhealthy conditions, in which basic health care is practically impossible; in this regard, there was also increasing work informality in several countries. ^3^ Government actions can be decisive in coping with these issues ^5^ ; there must be joint efforts by the government, the private sector, and individuals to reduce the economic impact of the pandemic. Some possible measures are suspension of taxes and establishment of universal basic income for the most vulnerable populations. ^18 , 19^

The pandemic is causing a period of economic recession in many countries, causing a complex pattern of health crises with worsening mental health, and increasing the number of homicides and suicides; it is known that low income also increases psychosocial stress. ^20^ In this context, it is possible that substance use may increase among the population. ^3^ Additionally, there are concerns about scarcity of supplies, which have led to irrational hoarding behaviors, and concerns about the significant financial losses also cause damage to psychological well-being. ^21^

#### Health systems

Health systems in several countries are suffering due to the excess demand caused by the pandemic. Effective planning to reconfigure services in the face of escalating demand is vital when dealing with this situation. Implementation of social isolation is also therefore important to avoid overburdening the health system. Planning should be based on the assumption that the majority of the population can contract the virus with little or no long-term effects, while using vital secondary health care resources to treat the small percentage of people who become seriously ill. ^22^ Many countries are using a combination of containment and mitigation activities with the intention of delaying large outbreaks of patients and leveling demand for hospital beds, while protecting the most vulnerable from infection, including the elderly and those with comorbidities. ^23^

## Interpersonal aspects

In this category we integrate issues related to family relationships, violence, and mental health.

### Family relationships

Changes in family relationships and routines due to social isolation have an impact on mental health and psychological well-being. ^24 , 25^ During home quarantine, family members spend most of the time together, which can cause or exacerbate tensions. Additionally, closing schools can increase stress for parents with school-aged children, who now have to support their children’s academic activities in addition to their children’s recreational activities and their other domestic and work chores. ^3^

### Violence

New Zealand’s experience has shown that family violence (including personal domestic violence, child abuse, and abuse of the elderly) can increase during and after large-scale disasters or crises. ^26^ A very recent note published in The Guardian reported increased incidence of violence in several different countries, with rises of 40 to 50% in Brazil and 25% in the United Kingdom and 20% in the first days of the confinement period in Spain. ^27^ Furthermore, social isolation from the pandemic may increase the risk of child abuse; this risk assessment is based on the increase in child abuse during school holidays, which also worsens during natural disasters, such as hurricanes. ^28^

According to ONU Mulheres (the Brazilian chapter of UN Women) ^29^ there is also a greater risk of violence against women in this pandemic period, during which victims are usually confined with the perpetrators of violence and often do not report the aggressions suffered. There is a growing rate of cases and therefore specialized support services such as shelters and health and assistance services (economic support packages) should be implemented as a matter of urgency. “ISA.bot” (a robot programmed to inform and welcome in cases of domestic or online violence that was launched in late 2019) has been updated in Messenger and Google Assistant to assist with safety and provide guidance for women. ^29^

In the face of these situations of violence, it is therefore important to think critically about the idealized representations of home and family; enabling listening places for people; asking people directly - on repeated occasions - if they feel safe at home; and offering protected and resourceful health services. The authors point out the need for governments around the world to allow these services to remain open. ^30^

### Mental health

From a psychopathological point of view, the pandemic is being considered a new trauma by mental health professionals, ^31^ comparable to other natural disasters such as earthquakes or tsunamis. ^32^ Specifically, social isolation decreases face-to-face connections and workaday social interactions, which is considered a relevant stressor. ^6 , 33 , 34^ Increased loneliness and reduced social interactions are well-defined risk factors for mental disorders, including schizophrenia and major depression. ^35^ It had already been stated that loneliness itself has become a modern epidemic; now, with the COVID-19 pandemic, loneliness has increased and is compounded by financial insecurity and the possibility of the death of a friend or family member. ^36^

Some studies indicate increases in stress disorders, such as Acute Stress Disorder (ASD) and Post Traumatic Stress Disorder (PTSD), and in emotional disorders such as depression, suicide and sleep disorders. ^6 , 37 - 41^ After about two months of pandemic, one survey showed an increase in symptoms of depression (16.5%), anxiety (28.8%), and stress (28.8%) in the general population ^8^ and, in particular, in health professionals. ^42^ Additionally, there were also increases in suicide rates linked to the psychological impact of COVID-19 in South Korea ^43^ and in India. ^44^ A study with 643 Canadian mothers of children aged 0 to 8 years in quarantine revealed that prevalence rates of maternal depressive symptoms and anxiety increased significantly with the age of the children. ^28^

Additionally, the effects of social isolation have had an impact on increased substance use. People being treated for a variety of conditions, such as alcoholism and other substance abuse, may experience additional complications from social isolation. ^11^ The effects of isolation also include PTSD symptoms, confusion, and anger. ^6 , 40^ Fear of contamination affects psychological well-being, because of the rapid spread and since there is considerable ignorance of the nature of the virus. ^11 , 45 , 46^ There is also fear for the future, which generates uncertainty and a lot of insecurity, also linked to the rise of fake news about the pandemic. ^35 , 38^ Uncertainty about contamination and death or about infecting family and friends can also cause dysphoric mental states. ^41^

Individual coping strategies include different activities that provoke pleasant mood and improve quality of life to overcome existential adversities, such as hobbies, physical exercise, reading, films, meditation, prayers, home maintenance, strengthening (or not) family bonds, studying, and listening to music, ^5 , 47^ in addition to interventions based on arts and life skills. ^38^ It has also been suggested that sources of information about the pandemic be limited, and a regular routine be maintained, mainly in terms of sleeping and eating, and that help should be requested when needed. ^9 , 35^ It is important to ensure that basic supplies (such as food, water, and medical supplies) are accessible, to reinforce a sense of altruism, ^6^ and to pay attention to one’s own needs, feelings, and thoughts. ^25^

## Use of technology

One of the measures employed to make isolation feasible is implementation of remote working, with online activities and revision of priorities (activities that do not require travel). There is an incentive to engage in intentional activities during this period to minimize the effect of isolation, for which use of technology is essential. ^18^ Additionally, increased use of technology to access online gaming platforms has also been observed, ^48^ and there have been increases in online gambling. ^3^

New health surveillance technologies have evolved with the pandemic, using machine-learning analysis for data collection, selection, and interpretation. For example, digital contact tracking is performed by analyzing people’s virtual accesses, identifying potentially infected people, tracking their contacts, and assessing social distancing. ^49^ Other examples include Google’s and Facebook’s initiatives for tracking infected people. ^50^ On the other hand, ethical issues are raised by health surveillance and it is considered a dilemma of safety versus privacy.

With regard to psychological care in the midst of a pandemic, a number of different modalities are offered. There are psychological listening channels via phone call or online platforms. ^9 , 51 , 52^ In the Brazilian scenario, formal psychological care remains online ^53 , 54^ or, when proven necessary, in person. ^51 , 55^ There is also an offer of psychological help through structured letters. ^56^ However, it is necessary to consider that a portion of the population has limited access to the internet, which limits the possibility of offering them support at this time. Furthermore, even if there is access, it is possible that there may also be difficulties with using smartphones or computers, as with the elderly ^25^ and it is therefore recommended that psychological services be provided via telephone in these cases. ^21 , 54^

## Discussion

The review conducted in this study identified several forms of impairment of people’s mental health. Most people have symptoms of distress, in addition to the fact that patients with pre-existing mental disorders are at risk of their clinical condition worsening due to suspension or reduction of activities at health centers. The psychological damage resulting from isolation stands out. It has been referred to as the “parallel pandemic” and will need specific coping strategies. ^37^

Modification of the form of care delivery to mental health patients is a consequence of the reality of social distance. ^35^ In Brazil, there was a great decrease in the flow of patients seen at mental health care centers run by the Unified Health System (SUS) due to the impact of social distance measures. One example of this situation is the fall in numbers of people presenting at Psychosocial Care Centers (CAPS) due to the lack of mobility, with a consequent report of increased stress, anxiety, and disorganization of routines, with changes in eating and sleeping. ^17^ In this context, psychoeducational interventions are revealed as important strategies, such as booklets and informative materials in general, ^8 , 57^ monitoring fake news, providing alternative support and service channels, and stimulating research to target prevention and mental health care policies. ^25^

Xiang et al. ^58^ suggest that three main factors should be included in government mental health strategies during COVID-19: 1) multidisciplinary mental health teams; 2) clear communication about the outbreak of COVID-19 and; 3) establishment of safe psychological counseling services (via electronic devices or applications). Additionally, protocols for managing stress, trauma, depression, and risky behavior need to be developed and available to guide professionals, covering areas relevant to the individual and collective mental health of the population. ^25^

Redelmeier & Shafir ^59^ point to awareness of pitfalls to combating the COVID-19 crisis, such as fear of the unknown, personal embarrassment (of adopting prevention strategies), and risk of neglect. A challenge during this period is to identify emergence or worsening of psychiatric disorders, which can be “invisible” and not easily detected by population screening. In this sense, the use of technology can facilitate access by isolated individuals to some types of health care, in addition to facilitating maintenance of work relationships and leisure activities.

For the population, it is essential to seek wellness strategies, identify triggers that cause psychological distress, keep in touch with the support network of family and friends (even if online), practice physical exercise, relieve stress, and avoid overexposure to media information related to the pandemic. It is necessary to reconcile leisure and work or study schedules with the quarantine. Public awareness campaigns should focus on ways to make quarantine easier and frictionless, rather than repeating reasons for staying indoors. ^60^

It is of note that only five Brazilian publications were identified in this review: three referring to economic effects and two on the use of technology as a way of adapting to this scenario. It is thus clear that the country is trying to adapt to the situation of isolation, but is very concerned with the economic effects, to the detriment of real health care of the population, as seen in European and North American countries.

This study has some limitations, inherent to its objective of conducting a review of recent literature on the effects of social isolation in the COVID-19 pandemic. Studies on the consequences of other recent epidemics (such as SARS in 2003 and African Ebola in 2014) were not considered. In order to obtain the greatest diversity of publications in a short period, letters to the editor and other review studies were included in this review.

Despite the recentness of the phenomenon of the COVID-19 pandemic, scientists are studying the theme in increasing depth, in a multidisciplinary and focused manner, in the search for better understanding of the current situation and its consequences, to facilitate responses to the pandemic and its repercussions. Greater support and a greater incentives for scientific production is expected, since only solid knowledge can explain and develop services that meet the demands and deliver comprehensive care to the population. Given this, it is necessary to plan an escalation of responses to the consequences of the pandemic, especially in view of the increased demand on the health sector and social services. The negative effects of social isolation can be prevented by public policies that offer a response to the economic recession, maintenance of social work, encouragement of quality care in mental health services, and community support for vulnerable families. Such measures, in the long run, will be decisive for the health for the population, especially its mental health.
